# Guild-level signature of gut microbiome for diabetic kidney disease

**DOI:** 10.1128/mbio.00735-24

**Published:** 2024-05-31

**Authors:** Shasha Tang, Guojun Wu, Yalei Liu, Binghua Xue, Shihan Zhang, Weiwei Zhang, Yifan Jia, Qinyuan Xie, Chenghong Liang, Limin Wang, Hongyan Heng, Wei Wei, Xiaoyang Shi, Yimeng Hu, Junpeng Yang, Lingyun Zhao, Xiaobing Wang, Liping Zhao, Huijuan Yuan

**Affiliations:** 1 Department of Endocrinology, Zhengzhou University People’s Hospital, Henan Provincial People’s Hospital, Henan Provincial Key Medicine Laboratory of Intestinal Microecology and Diabetes, Zhengzhou, China; 2 Department of Biochemistry and Microbiology and New Jersey Institute for Food, Nutrition, and Health, School of Environmental and Biological Sciences Rutgers University, New Brunswick, New Jersey, USA; 3 Rutgers-Jiaotong Joint Laboratory for Microbiome and Human Health, New Brunswick, New Jersey, USA; 4 State Key Laboratory of Microbial Metabolism and Ministry of Education Key Laboratory of Systems Biomedicine, School of Life Sciences and Biotechnology, Shanghai Jiao Tong University, Shanghai, China; University of Hawaii at Manoa, Honolulu, Hawaii, USA

**Keywords:** DKD, guild, gut microbiome

## Abstract

Current microbiome signatures for chronic diseases such as diabetic kidney disease (DKD) are mainly based on low-resolution taxa such as genus or phyla and are often inconsistent among studies. In microbial ecosystems, bacterial functions are strain specific, and taxonomically different bacteria tend to form co-abundance functional groups called guilds. Here, we identified guild-level signatures for DKD by performing in-depth metagenomic sequencing and conducting genome-centric and guild-based analysis on fecal samples from 116 DKD patients and 91 healthy subjects. Redundancy analysis on 1,543 high-quality metagenome-assembled genomes (HQMAGs) identified 54 HQMAGs that were differentially distributed among the young healthy control group, elderly healthy control group, early-stage DKD patients (EDG), and late-stage DKD patients (LDG). Co-abundance network analysis classified the 54 HQMAGs into two guilds. Compared to guild 2, guild 1 contained more short-chain fatty acid biosynthesis genes and fewer genes encoding uremic toxin indole biosynthesis, antibiotic resistance, and virulence factors. Guild indices, derived from the total abundance of guild members and their diversity, delineated DKD patients from healthy subjects and between different severities of DKD. Age-adjusted partial Spearman correlation analysis showed that the guild indices were correlated with DKD disease progression and with risk indicators of poor prognosis. We further validated that the random forest classification model established with the 54 HQMAGs was also applicable for classifying patients with end-stage renal disease and healthy subjects in an independent data set. Therefore, this genome-level, guild-based microbial analysis strategy may identify DKD patients with different severity at an earlier stage to guide clinical interventions.

**IMPORTANCE:**

Traditionally, microbiome research has been constrained by the reliance on taxonomic classifications that may not reflect the functional dynamics or the ecological interactions within microbial communities. By transcending these limitations with a genome-centric and guild-based analysis, our study sheds light on the intricate and specific interactions between microbial strains and diabetic kidney disease (DKD). We have unveiled two distinct microbial guilds with opposite influences on host health, which may redefine our understanding of microbial contributions to disease progression. The implications of our findings extend beyond mere association, providing potential pathways for intervention and opening new avenues for patient stratification in clinical settings. This work paves the way for a paradigm shift in microbiome research in DKD and potentially other chronic kidney diseases, from a focus on taxonomy to a more nuanced view of microbial ecology and function that is more closely aligned with clinical outcomes.

## INTRODUCTION

Diabetic kidney disease (DKD) is a prevalent and costly microvascular complication of diabetes (1). It has surpassed glomerulonephritis as the leading cause of chronic kidney disease (CKD) and end-stage renal disease (ESRD) (2, 3). Despite efforts to maintain stable blood sugar control, the progression from diabetes to DKD is often inevitable (4). DKD significantly increases the risk of cardiovascular disease and related mortality in diabetic patients, with an all-cause mortality rate remaining high even with active lifestyle interventions and multi-factorial measures such as blood sugar and blood pressure control (5–7). The lack of early diagnosis and effective treatment further exacerbates this problem (8). DKD typically presents with an insidious onset, and diagnostic kidney biopsies are seldom performed in diabetic patients (9). By the time symptoms become evident, significant proteinuria and progressive decline in glomerular filtration rate are frequently observed. Complications, such as metabolic acidosis, anemia, and electrolyte disorders, become more likely as the disease progresses (10). These complexities highlight the dynamic, multifactorial nature of DKD, involving genetic and environmental factors (11), and therefore underscore the need for more sensitive biomarkers for early DKD diagnosis and prediction to guide clinical interventions.

In recent years, increasing attention has been focused on the role of the gut microbiota in human health, with growing evidence suggesting its involvement in the pathogenesis of DKD (12, 13). Dysbiotic gut microbiota in DKD patients often exhibits an increase in pathogenic bacteria and a decrease in beneficial bacteria, resulting in an abnormal colony structure dominated by Gram-negative facultative anaerobic bacteria (14). The overexpression of Gram-negative bacteria stimulates the production of various antigens, such as lipopolysaccharides (LPS), leading to systemic inflammatory responses and renal damage (15, 16). Reductions in short-chain fatty acid (SCFA)-producing bacteria contribute to tubulointerstitial damage by disrupting cholesterol homeostasis, thereby promoting the occurrence and development of DKD (17). Additionally, the proliferation of urease-producing pathogens in DKD patients leads to increased ammonia concentration and pH in the intestinal cavity, enhancing intestinal permeability and the entry of gut-derived uremic toxins such as indoxyl sulfate (IS) into circulation, further inducing kidney damage (18). Previous research on high-fiber diet interventions in obese and diabetic patients has shown significant reductions in IS levels in urine and indoles in feces as the levels of indole and endotoxin-producing bacteria decreased and SCFA-producing bacteria increased (19, 20), suggesting that interventions targeting specific gut microbiota populations could be promising strategies for DKD prevention and treatment.

However, most studies on the relationship between DKD and the microbiota have primarily utilized “genus” or “phylum” taxonomic units to identify disease-related bacteria, resulting in inconsistent findings (21–23). For instance, a case-control study comparing the gut microbiota composition of DKD and non-DKD individuals identified 107 significantly increased bacterial genera in DKD patients, including *Bifidobacterium* (22), traditionally considered an important beneficial bacteria group lacking in the intestines of DKD patients (23). This highlights the controversy surrounding the use of taxonomic units for identifying disease-critical bacteria, which may yield false and misleading results. The lack of reproducible findings in these studies is attributed to the absence of consensus on identifying gut bacterial populations associated with health in different microbial ecosystems (24). Given the genetic diversity of bacterial strains and ecological interactions between strains, consolidating them into a single taxonomic unit may result in zero or false correlations with disease phenotypes (24). Furthermore, the classification of bacteria based on taxonomic units relies on the similarity of their DNA sequences to the nearest neighbor recorded in a reference database, leading to the exclusion of unclassified sequences from subsequent analysis, thereby limiting the exploration of key bacteria to existing databases and overlooking the potential importance of unknown bacteria.

To address these challenges, this study aims to analyze the ecological behavior of bacteria represented by different sequences, classify gut bacteria of different taxonomic statuses capable of cooperating with each other into ecological functional groups (guilds) (24), and explore key ecological functional groups, and their key functional genes involved in DKD development to enable early DKD identification and guide effective clinical prevention and treatment. Fecal samples were collected from 57 patients with early-stage DKD (estimated glomerular filtration rate [eGFR] ≥ 60 mL/min/1.73 m^2^), 59 patients with late-stage DKD (eGFR < 60 mL/min/1.73 m^2^), and healthy volunteers, including 46 individuals and 45 young healthy volunteers. Metagenomic sequencing was performed on these 207 samples, yielding 1,543 nonredundant high-quality metagenome-assembled genomes (HQMAGs) with completeness >95% and contamination <5%. Microbiome signature of 54 HQMAGs was identified, centered on guilds, with functions annotated at the genome level, and correlated with clinical indicators to construct bacterial functional groups related to DKD. Furthermore, these characteristics were utilized to differentiate between patients with different severities of DKD and between DKD patients and healthy populations, with their classification capabilities validated in an independent research data set.

## RESULTS

### Clinical characteristics among patients of differing DKD severities and healthy participants

Following stringent inclusion and exclusion criteria, stool samples and clinical data were obtained from 57 patients with early-stage diabetic kidney disease (EDG), 59 with late-stage diabetic kidney disease (LDG), alongside 46 older healthy controls (healthy control group [HCG]), and 45 younger healthy control group (young healthy control group [YHCG]). Upon characterizing their clinical parameters, gender distribution showed no significant variability across the four groups (*P* = 0.160, Pearson’s χ^2^ test; Table 1). There was no marked difference in body mass index (BMI), age, and the levels of HbA1c between EDG and LDG groups (*P* > 0.05, BMI and age: one-way analysis of variance (ANOVA] with Tukey’s post-hoc test; HbA1c: Kruskal-Wallis test followed by Dunn’s post-hoc test; Table 1). Within the healthy groups, eGFR decreased in the HCG group compared with the YHCG group, accompanied by increased levels of TG and lactate dehydrogenase (LDH; *P* < 0.05, the Kruskal-Wallis test followed by Dunn’s post-hoc test; Table 1). As disease severity intensified from the YHCG/HCG to the EDG/LDG groups, with eGFR declininggradually, there was a substantial escalation in markers of renal impairment such as urinary albumin-to-creatinine ratio (UACR), creatinine (CREA), uric acid (UA), cystatin C (CysC), and retinol-binding protein (RBP). In parallel, indicators of myocardial injury like LDH and creatine kinase (CK) also showed a significant uptick. Conversely, serum albumin (ALB) levels demonstrated a notable reduction (*P* < 0.001, one-way ANOVA; Table 1).

**TABLE 1:** Clinical characteristics comparison across different severities of diabetic kidney disease and healthy participants^*a*^

Variable	YHCG (*n* = 45)	HCG (*n* = 46)	EDG (*n* = 57)	LDG (*n* = 59)	*P*
Age (years)	28.6 ± 4.7^a^	54.6 ± 6.2^b^	57.3 ± 9.5^bc^	60.9 ± 9.4^c^	<0.001
Male [*n* (%)]	31 (68)^ab^	27 (59)^a^	45 (79)^b^	42 (71)^ab^	0.160
BMI (kg/m^2^)	23.4 ± 3.7^a^	25.5 ± 3.5^b^	25.8 ± 3.2^b^	24.7 ± 3.3^ab^	0.004
eGFR (mL/min)	121.6 (117.0, 127.1)^a^	102.2 (97.0, 108.8)^b^	98.4 (84.1, 106.7)^b^	17.5 (10.4, 39.1)^c^	<0.001
UACR (mg/g)	3.0 (2.1, 4.6)^a^	4.1 (1.7, 7.0)^a^	362.1 (86.6, 986.4)^b^	2,040 (814.3, 4317)^c^	<0.001
CREA (μmol/L)	68.5 ± 11.8^a^	64.4 ± 12.1^a^	75.7 ± 18.7^a^	277 (152, 440)^b^	<0.001
UA (μmol/L)	345.5 ± 102.9^a^	312.5 ± 67.0^a^	335.3 (297.0, 389.6)^a^	408.0 ± 108.9^b^	<0.001
Cys C (mg/L)	0.8 ± 0.1^a^	0.8 ± 0.1^a^	1.1 ± 0.2^b^	3.0 ± 1.2^c^	<0.001
RBP (mg/L)	36.7 ± 7.0^a^	40.3 ± 9.6^a^	53.1 ± 15.1^b^	73.9 ± 20.8^c^	<0.001
HbA1c (%)	5.0 ± 0.3^a^	5.3 (5.1, 5.5)^a^	9.4 ± 1.9^b^	7.2 (6.5, 8.4)^b^	<0.001
TG (mmol/L)	1.1 (0.8, 1.3)^a^	1.6 (1.0, 2.1)^b^	1.9 (1.2, 3.4)^b^	1.6 (1.1, 2.4)^b^	<0.001
TC (mmol/L)	4.3 (3.8, 4.8)^a^	4.8 (4.1, 5.3)^a^	4.6 (4.3, 5.8)^a^	4.1 (3.2, 5.7)^a^	0.011
LDL-C (mmol/L)	2.2 (1.9, 2.6)^ab^	2.6 (2.2, 3.0)^ab^	2.7 (2.1, 3.1)^a^	2.0 (1.6, 2.9)^b^	0.007
HDL-C (mmol/L)	1.3 (1.1, 1.5)^a^	1.1 (1.1, 1.5)^a^	1.0 (0.8, 1.2)^b^	0.9 (0.8, 1.1)^b^	<0.001
LDH (U/L)	103.4 (74.5, 136.9)^a^	141.3 (115.6, 173.1)^b^	196.2 (177.8, 220.3)^c^	230.3 (193.3, 272.5)^d^	<0.001
CK (U/L)	87.0 (62.4, 107.1)^a^	79.2 (54.1, 104.7)^a^	73.3 (58.1, 118.5)^a^	110.3 (81.6, 190.0)^b^	<0.001
ALT (U/L)	14.4 (9.6, 26.9)^a^	18.4 (13.3, 25.5)^ab^	20.8 (14.6, 31.2)^b^	15.3 (10.5, 22.2)^a^	0.004
AST (U/L)	18.6 (15.6, 22.8)^ab^	21.4 (17.9, 23.8)^a^	19.6 (14.0, 26.0)^ab^	16.0 (12.5, 21.1)^b^	0.002
ALB (mg/L)	47.6 ± 3.2^a^	45.3 ± 2.3^a^	39.8 ± 4.8^b^	35.0 ± 6.4^c^	<0.001

^*a*^ This table presents a comparative analysis of clinical parameters among individuals with varying severities of DKD and healthy controls. The values are reported as either mean ± SD, median along with the interquartile range (IQR), or as a count where applicable. The distribution assumption for normality of the variables was tested using the Kolmogorov-Smirnov test. Based on the outcomes of this test, statistical differences among groups were determined using one-way ANOVA with Tukey’s post-hoc test for age, BMI, Cys C, RBP, and ALB. Gender distribution was assessed using Pearson’s χ^2^ test. The Kruskal-Wallis test followed by Dunn’s post-hoc test was applied for the analysis of all other indicators. *P* values were adjusted for multiple comparisons using Dunn’s test. Different superscripted symbols (a, b, c, and d) in a row indicated that the means or medians of the different groups are significantly different (adjusted *P* < 0.05). Abbreviations: HbA1c, glycated hemoglobin; TG, triglycerides; TC, total cholesterol; LDL-C, low-density lipoprotein cholesterol; HDL-C, high-density lipoprotein cholesterol; ALT, alanine aminotransferase; and AST, aspartate aminotransferase.

### Structural changes in the gut microbiome linked with varying degrees of DKD severity

Metagenomic sequencing was performed on 207 samples to profile the gut microbiome of the participants. We achieved strain-/subspecies-level resolution by *de novo* reconstructing 1,543 nonredundant HQMAGs, merging any two HQMAGs into one if their average nucleotide identity surpassed 99%. These HQMAGs represented over 76.77% ± 5.69% (mean ± SD) of the total sequenced reads and served as the foundational variables for further analysis.

**Fig 1:**
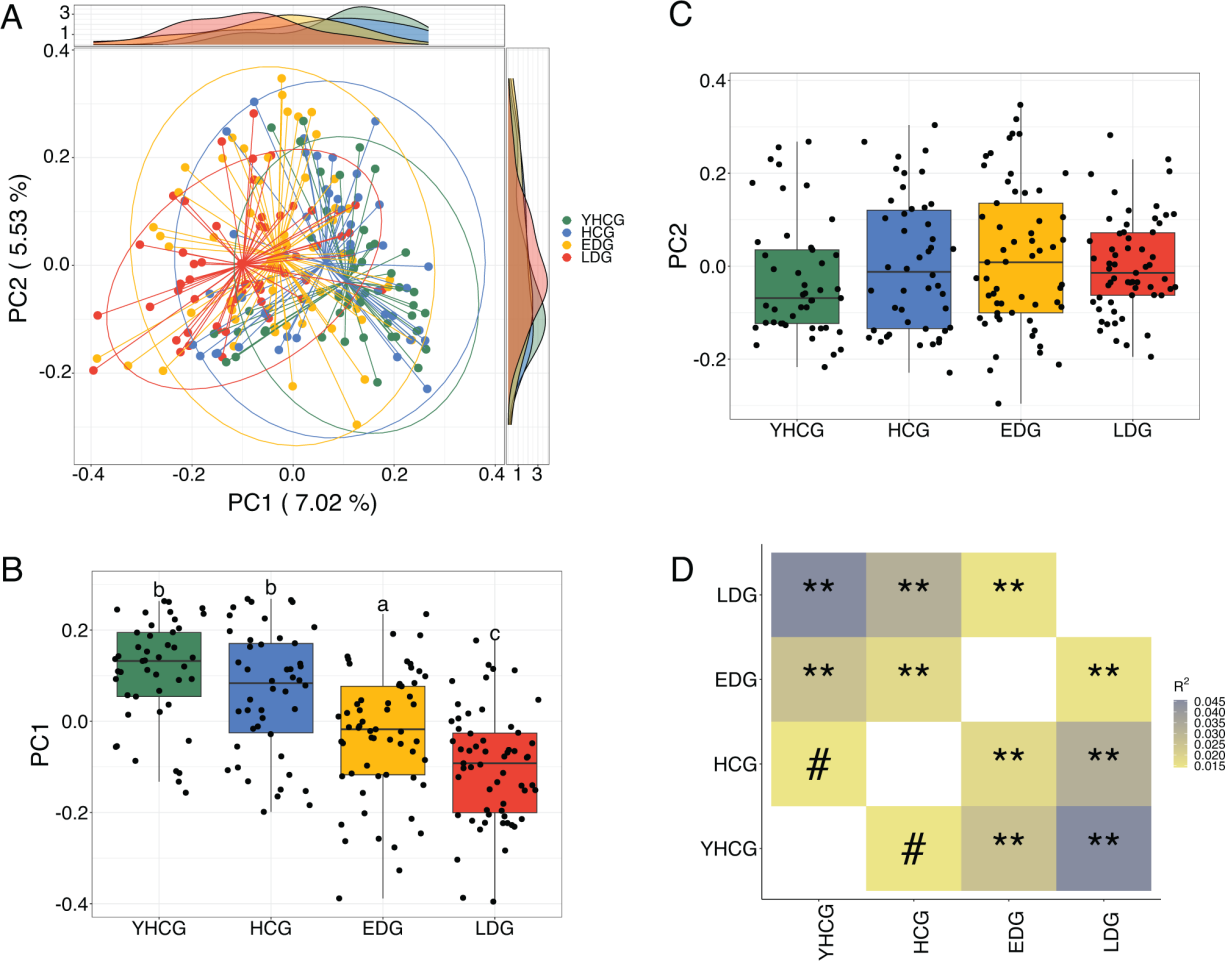
Variations in the overall structure of the gut microbiota associated with DKD and disease severity. (**A**) PCoA based on the Bray-Curtis distances calculated from 1,543 genomes, illustrating the distribution and clustering of the gut microbiota among the study groups. (**B** and **C**) Comparative analysis of the PC1 and PC2 scores across the groups. Box plots represent the median values and interquartile ranges (IQRs), with whiskers extending to the lowest and highest values within 1.5 times the IQR from the first and third quartiles, respectively. Outliers are depicted as individual points. The Kruskal-Wallis test followed by Dunn’s post hoc test (two-sided) was employed for group comparisons. Compact letter displays denote the significance as per the post hoc test, with Benjamini-Hochberg (BH) adjusted *P*-values: *P* < 0.05 deemed significant. (**D**) Pairwise PERMANOVA testing among the four groups, with # indicating BH adjusted *P* < 0.1 and ** indicating BH adjusted *P* < 0.01. The groups are YHCG (*n* = 45), HCG (*n* = 46), early-stage DKD (EDG, *n* = 57), and late-stage DKD (LDG, *n* = 59).

Regarding beta-diversity, based on Bray-Curtis distance, principal coordinate analysis (PCoA) revealed distinct separations in the gut microbiota structure along PC1 (Fig. 1A through C). This separation aligned with the division of the four groups when considering 17 different clinical parameters (Fig. S1). A single-factor permutational multivariate analysis of variance (PERMANOVA) test showed that both age (*R*² =0.0169, *P* = 0.001) and group classification (*R*² =0.0378, *P* = 0.001) were significantly associated with the overall gut microbiota composition. Nevertheless, a marginal PERMANOVA test suggested that while group classification maintained a significant relationship with the microbiota structure after adjusting for age (*R*² =0.0254, *P* = 0.001), the influence of age was not significant when controlling for group classification (*R*² =0.0045, *P* = 0.561). This suggests that within our data set, certain variations in gut microbiota were uniquely associated with the specific groups, independent of age. Pairwise PERMANOVA comparisons between the four groups showed that the microbiota of the control groups significantly differed from that of the disease groups (Fig. 1D; HCG vs EDG adjusted *P* = 0.0036, HCG vs LDG adjusted *P* = 0.002, YHCG vs EDG adjusted *P* = 0.002, and YHCG vs LDG adjusted *P* = 0.002). A comparison with controls showed that microbiota dissimilarity augmented with increasing disease severity, as indicated by a larger *R*² value in comparisons between HCG/YHCG and LDG than HCG/YHCG and EDG. Additionally, we noted a significant difference in gut microbiota structure between EDG and LDG groups (adjusted *P* = 0.002) and a marginally significant difference between YHCG and HCG groups (adjusted *P* = 0.083). These findings demonstrate that the overall structure of the gut microbiota is associated with clinical parameters and the extent of disease severity.

### Two competing guilds in association with DKD and its severity

**Fig 2:**
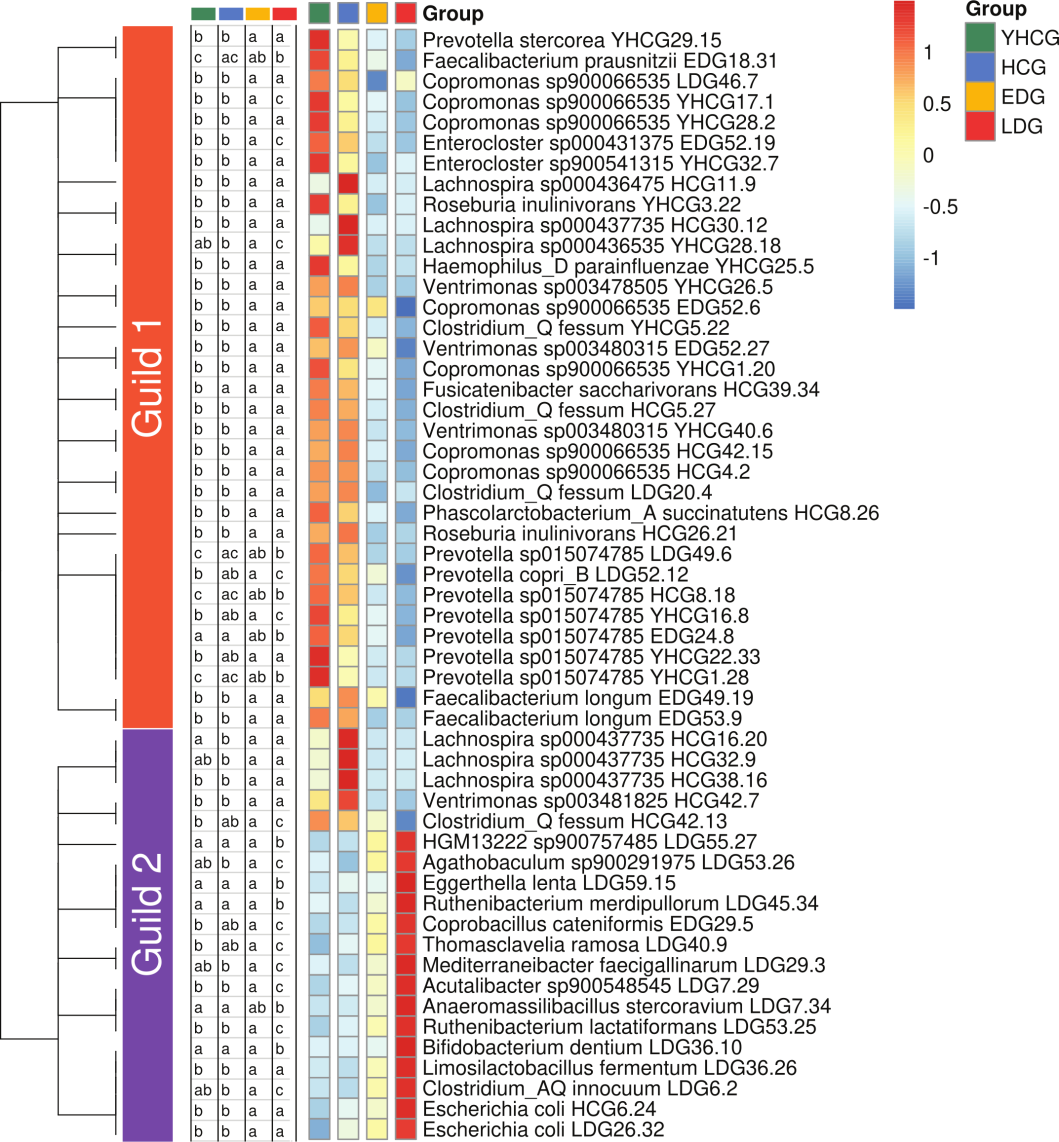
Differentially abundant gut microbiota genomes interacted with each other and organized as two guilds. Heatmap of the 54 HQMAGs identified by RDA and showing differences between the four groups. RDA was conducted based on the Hellinger transformed abundance of all the HQMAGs and used group assignment of samples as an environmental variable. HQMAGs with at least 10% of the variability in their abundance explained by constrained axes were selected. A Kruskal-Wallis test followed by Dunn’s post hoc test (two-sided) was used to test the differences between groups. Compact letters indicate the significance of the post hoc test (Benjamini-Hochberg adjusted *P* < 0.05 is significant). The heatmap shows a mean abundance of each HQMAG in each group. The mean abundance was further scaled across each row. The genomes were ordered based on their positions in the hierarchical clustering tree, which was calculated from complete linkage and co-abundance correlations. The co-abundance correlation was calculated using Fastspar (*n* = 207 subjects). All significant correlations with *P* ≤ 0.001 were included. WGCNA was used to identify the two guilds.

**Fig 3:**
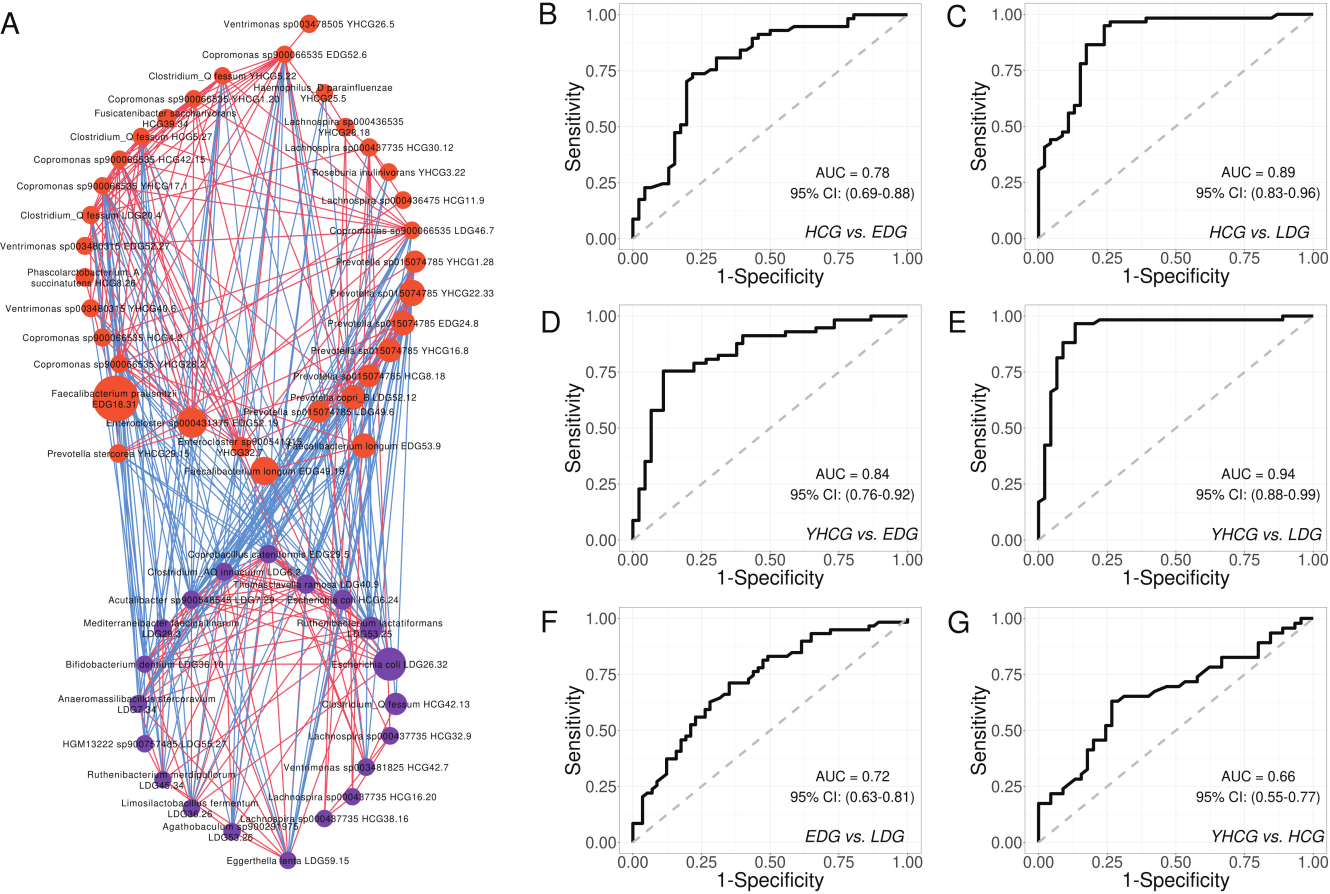
Members of the two guilds as microbiome signature for DKD. (**A**) Co-abundance network of the HQMAGs in the two guilds. Red and blue colors indicate all significantly positive and negative correlations, respectively. Node size indicates the average abundance of the HQMAGs in 207 samples. Node color indicates guild: guild 1, orange; guild 2, purple. (**B–G**) Based on the abundance matrix of the 54 HQMAGs, random forest classification models with leave-one-out cross validation were trained to classify groups. The area under the receiver operating characteristic (AUROC) is shown here.

In our quest to pinpoint the microbial constituents linked with DKD, redundancy analysis (RDA) was employed, taking group classification as the constraining variable. Among the 1,543 HQMAGs, 54 were found to have over 10% of their variance accounted for by this variable (Fig. S2). These 54 HQMAGs spanned 23 different genera, with their abundances presenting significant variations across the four groups (Fig. 2, Kruskal-Wallis test followed by Dunn’s post hoc, adjusted *P* < 0.05). Considering that bacteria in the gut ecosystem do not operate solitarily but form cohesive functional assemblies, or “guilds,” that interact and impact host health (24), we engaged in co-abundance analysis of these 54 HQMAGs. Connected component clustering analysis, only considering all significant correlations regardless of direction, revealed that the 54 HQMAGs formed one highly interconnected unit. To identify potential guild configurations while accounting for correlation directions, we additionally employed hierarchical clustering and weighted correlation network analysis (WGCNA) (25). The 54 HQMAGs clustered into two distinct guilds (Fig. 2). Guild 1 comprised eight HQMAGs from *Prevotella*, seven from *Coprococcus*, three each from *Clostridium fessum*, *Lachnospira*, and *Veillonella*, two each from *Enterocloster*, *Faecalibacterium longum*, and *Roseburia inulinivorans*, and one each from *Faecalibacterium prausnitzii*, *Fusicatenibacter saccharivorans*, *Phascolarctobacterium succinatutens*, and *Haemophilus parainfluenzae*. Guild 2 consisted of three HQMAGs from *Lachnospira*, two from *Escherichia coli*, and one from each of 15 different taxa. Members within each guild displayed positive interconnections, whereas 93% of significant interactions between the two guilds were negative (Fig. 3A). These results suggest that the differential genomes between the groups were connected and may work as cohesive functional assemblies.

To ascertain whether the members in the two guilds could distinguish DKD from controls, Random Forest classifiers using the abundance of the 54 HQMAGs were constructed (Fig. 3B through E). Receiver operating characteristic (ROC) curve analyses revealed that these genomes had a moderate to good diagnostic ability to differentiate HCG from EDG (area under the receiver operating characteristic [AUROC] = 0.78) and HCG from LDG (AUROC = 0.89), and a good to excellent ability to separate YHCG from EDG (AUROC = 0.84) and YHCG from LDG (AUROC = 0.94). Moreover, these genomes demonstrated a moderate proficiency in classifying patients with different levels of DKD severity, i.e., EDG vs LDG, as indicated by an AUROC of 0.72 (Fig. 3F). The capacity to distinguish YHCG from HCG was less pronounced, with an AUROC of 0.66 (Fig. 3G), consistent with the subtler dissimilarity in overall structure between these two groups.

**Fig 4:**
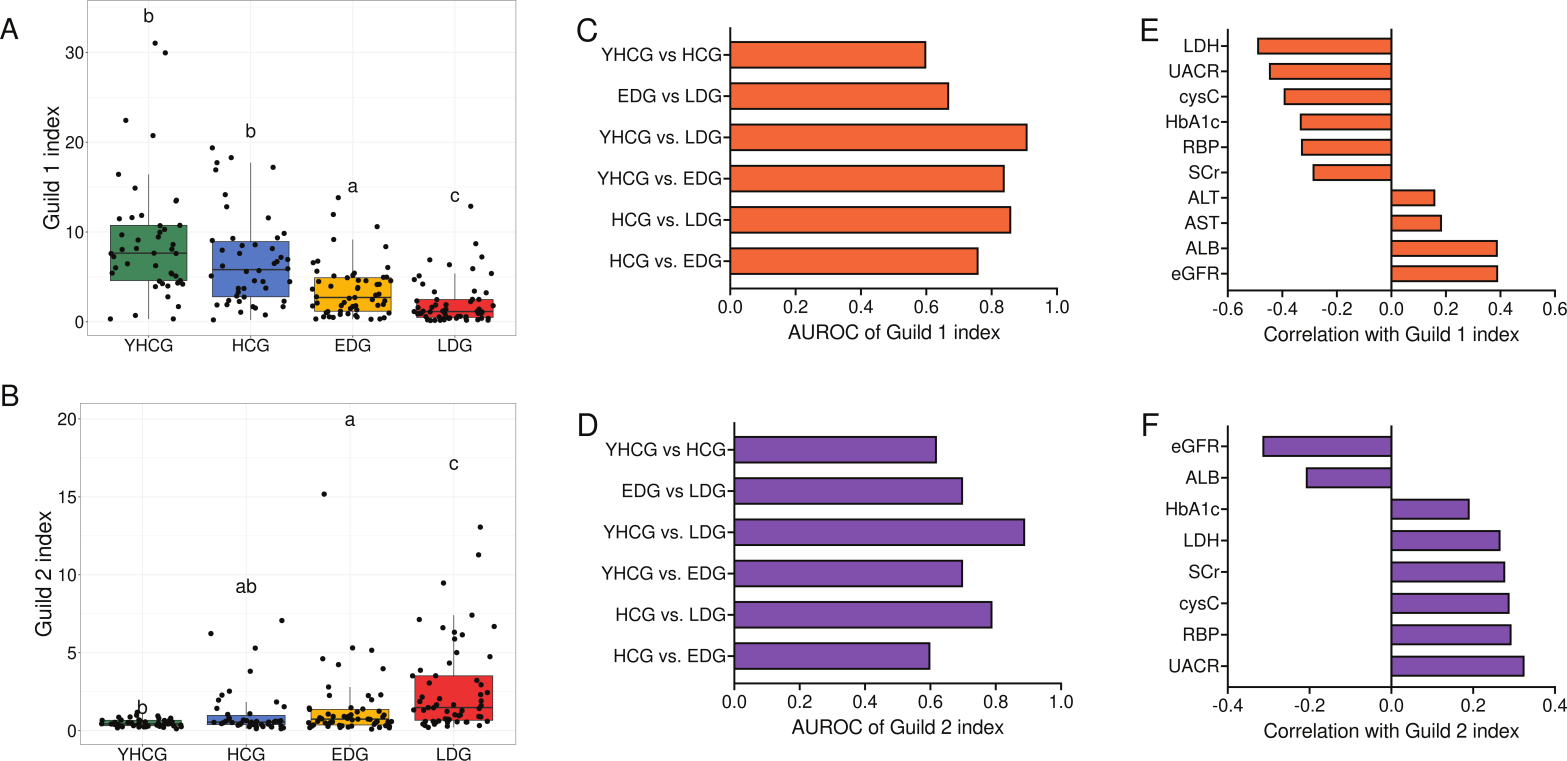
Guild indices as microbiome signature for DKD and are correlated with a wide range of clinical parameters. (**A** and **B**) Comparison of the guild 1 index and guild 2 index between the groups. $Guild\;index_{j}=\sum_{i\in N}A_{ij}\;\times \;Simpson^{\prime }s\;diversity\;index_{j}$, where *A*_*ij*_ is the abundance of HQMAG *i* in the sample *j* and *N* is the HQMAGs in the guild. Boxes show medians and interquartile ranges (IQRs); whiskers denote the lowest and highest values that were within 1.5× the IQR from the first and third quartiles, and outliers are shown as individual points. Kruskal-Wallis test followed by Dunn’s post hoc test (two-sided) was used to compare groups. Compact letters indicate the significance of the post hoc test (Benjamini-Hochberg adjusted *P* < 0.05 is significant). YHCG, *n* = 45; HCG, *n* = 46; EDG, *n* = 57; LDG, *n* = 59. (**C** and **D**) The bar plots show the AUROC. (**E** and **F**) The bar plots show the correlation between the guild indices and clinic parameters. Age-adjusted partial Spearman’s correlation was calculated. Correlations with Benjamini-Hochberg adjusted *P* < 0.05 are shown.

In addition to apply machine-learning models with the 54 HQMAGs in a black box way, we sought to aggerate the 54 HQMAGs based on their guild pattern to examine the guild-level associations between gut microbiota and host health. We then computed a guild index from the abundance and Simpson’s diversity of the HQMAGs in each guild. Compared to controls, the guild 1 index was markedly lower in both EDG and LDG groups, declining further with increased disease severity (Fig. 4A). In contrast, the guild 2 index was elevated in DKD patients and intensified with disease progression (Fig. 4B). Indices for both guilds were similar between YHCG and HCG. Subsequent ROC curve analyses were conducted to validate the connection between the guild indices and DKD, assessing their classification efficacy among different groups (Fig. 4C and D). Both guild indices demonstrated moderate to an excellent capability in distinguishing between YHCG and EDG/LDG, as well as between HCG and LDG (guild 1 index: YHCG vs EDG, AUROC = 0.84; YHCG vs LDG, AUROC = 0.91; HCG vs LDG, AUROC = 0.86; guild 2 index: YHCG vs EDG, AUROC = 0.70; YHCG vs LDG, AUROC = 0.89; HCG vs LDG, AUROC = 0.79). The guild 1 index outperformed the guild 2 index in classifying HCG from LDG (AUROC = 0.76 vs 0.60) but was less effective in differentiating EDG from LDG (AUROC = 0.67 vs 0.70). In the comparison between YHCG and HCG, both guild indices exhibited lower performance (guild 1 index, AUROC = 0.60; guild 2 index, AUROC = 0.62). Overall, these outcomes suggest the potential of utilizing the guild index as a biomarker to differentiate between DKD and healthy controls, as well as to gauge DKD severity. Correlation analyses between guild indices and clinical parameters were conducted to further elucidate the relationships between gut microbiota and host health. eGFR showed significant correlations (partial Spearman’s correlation; Benjamini-Hochberg adjusted *P* < 0.05) with both guild indices—positive with guild 1 and negative with guild 2 (Fig. 4E and F). Additionally, guild 1 index correlated negatively with LDH, UACR, cysC, HbA1c, RBP, and SCr and positively with ALB, AST, and ALT. Conversely, guild 2 index correlated positively with UACR, RBP, cysC, SCr, LDH, and HbA1c and negatively with ALB. These results underscore the associations of the two guilds with a broad spectrum of clinical parameters, highlighting their potential as indicators of overall health status and disease progression.

### Guild-specific functional disparities among the two competing bacterial guilds

**Fig 5:**
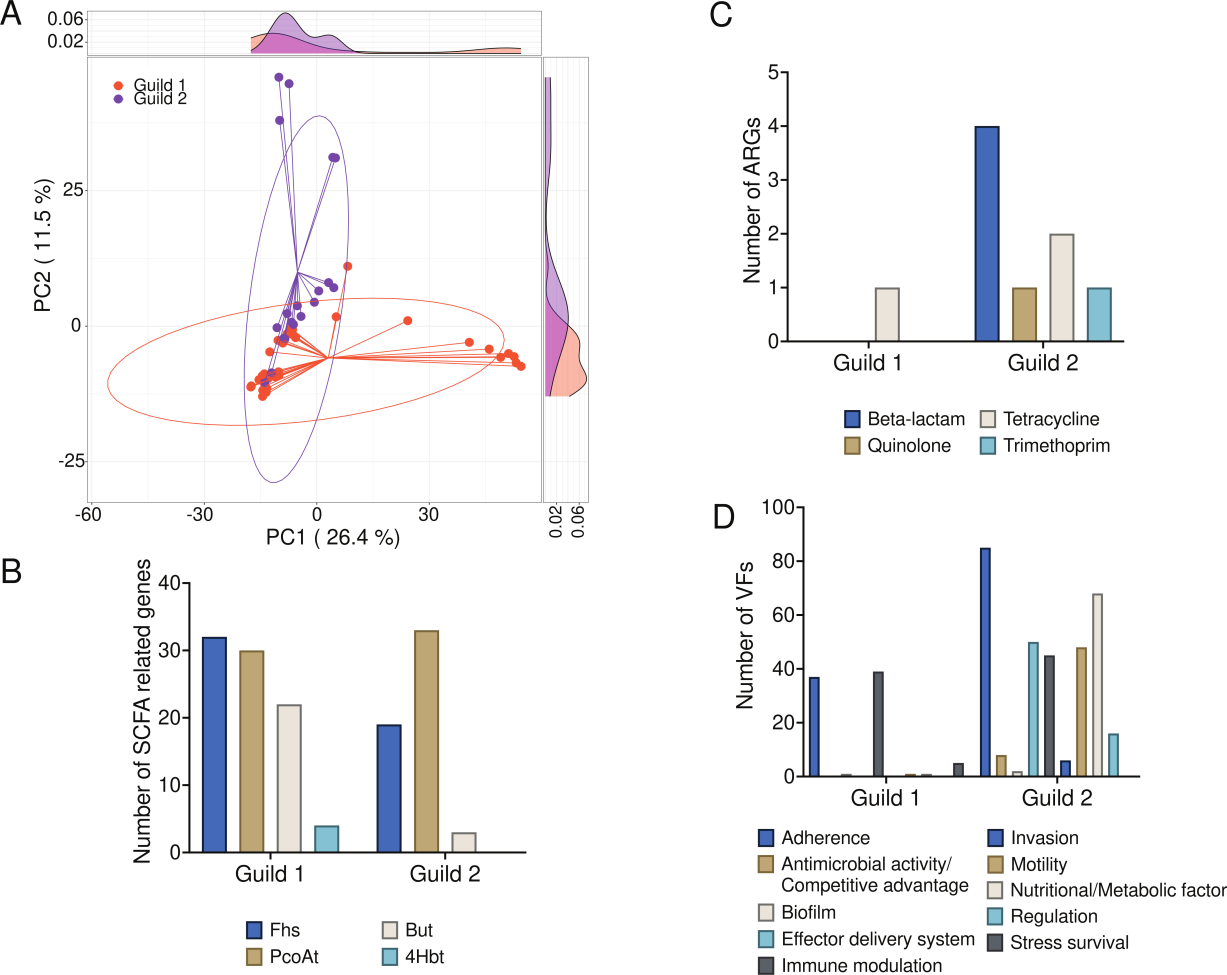
Functional genetic distinction between the two guilds. (**A**) PCoA based on Euclidean distance calculated from copy numbers of KO in the 54 genomes. (**B**)The bar plot shows the copy number of SCFA-producing genes. (**C**)The bar plot shows the number of ARGs and the corresponding antibiotic resistance types. (**D**)The bar plot shows the number of genes encoding VF and classes of VFs.

In pursuit of understanding the genetic underpinnings of the associations between the two guilds and host health, we undertook a genome-centric functional analysis of the HQMAGs within each guild. An exhaustive functional annotation of Kyoto Encyclopedia of Genes and Genomes (KEGG) Orthologs (KOs) for each genome was performed. In total, 2,935 and 4,329 KOs were found in guild 1 and guild 2, respectively. The PCoA plot, based on Euclidean distances derived from KO copy numbers, depicted a marked distinction between the two guilds (Fig. 5A, PERMANOVA *P* = 0.003). KOs in guild 1 and guild 2 were mapped to 223 and 251 KEGG modules (Fig. S3). Guild 1 uniquely harbored seven KOs such as M00623 which generates protocatechuate. Protocatechuate has been reported to have an antiglycative effect in kidneys (26). Thirty-five unique modules were observed in guild 2. Among the 35 unique modules, 9 were antibiotic resistance-related modules, 1 was related to LPS biosynthesis, and 1 was related to benzoate production. Benzoate is the microbial precursor of hippurate, which is a harmful uremic toxin and is increased in end-stage DKD patients (27). Notably, several other metabolites produced by the gut microbiota, such as SCFAs and indoles, along with their derivatives, have been implicated in DKD (28). Focusing on the terminal genes of butyrate biosynthesis pathways (e.g., but, buk, atoA/D, and 4Hbt), HQMAGs in guild 1 manifested significantly elevated copy numbers of but (Fig. 5B, *P* = 0.018) and a greater number of genomes containing but (14/34 vs 2/20, Fisher’s exact test *P* = 0.029). There was also a trend for guild 1 to possess more 4Hbt genes than guild 2. Other genes involved in butyrate synthesis were absent in HQMAGs from both guilds. Guild 1’s HQMAGs were more likely to harbor propionate production genes compared to those in guild 2 (23/30 vs 4/29, *P* = 0.0016). There was no discernible difference in acetate production genes between the two guilds. Guild 1’s HQMAGs did not contain genes for tryptophanase, which converts tryptophan to indole, whereas two *E. coli* genomes in guild 2 did. Regarding antibiotic resistance genes (ARGs), one genome in guild 1 encoded one ARG, whereas four genomes in guild 2 encoded eight ARGs (Fig. 5C). From a pathogenicity standpoint, 73.53% of HQMAGs in guild 1 encoded virulence factor (VF) genes, totaling 84 VFs across 6 VF categories. In contrast, 95% of HQMAGs in guild 2 encoded VF genes, encompassing 328 VFs across 9 VF categories (Fig. 5D). Collectively, these findings demonstrate distinct genetic capabilities between the two guilds, with guild 1 exhibiting a more beneficial profile and guild 2 showing more detrimental characteristics. Hence, the genetic disparities between the two guilds could elucidate their differential associations with DKD and its severity.

### The microbiome signature of two competing guilds validated in an independent data set

**Fig 6:**
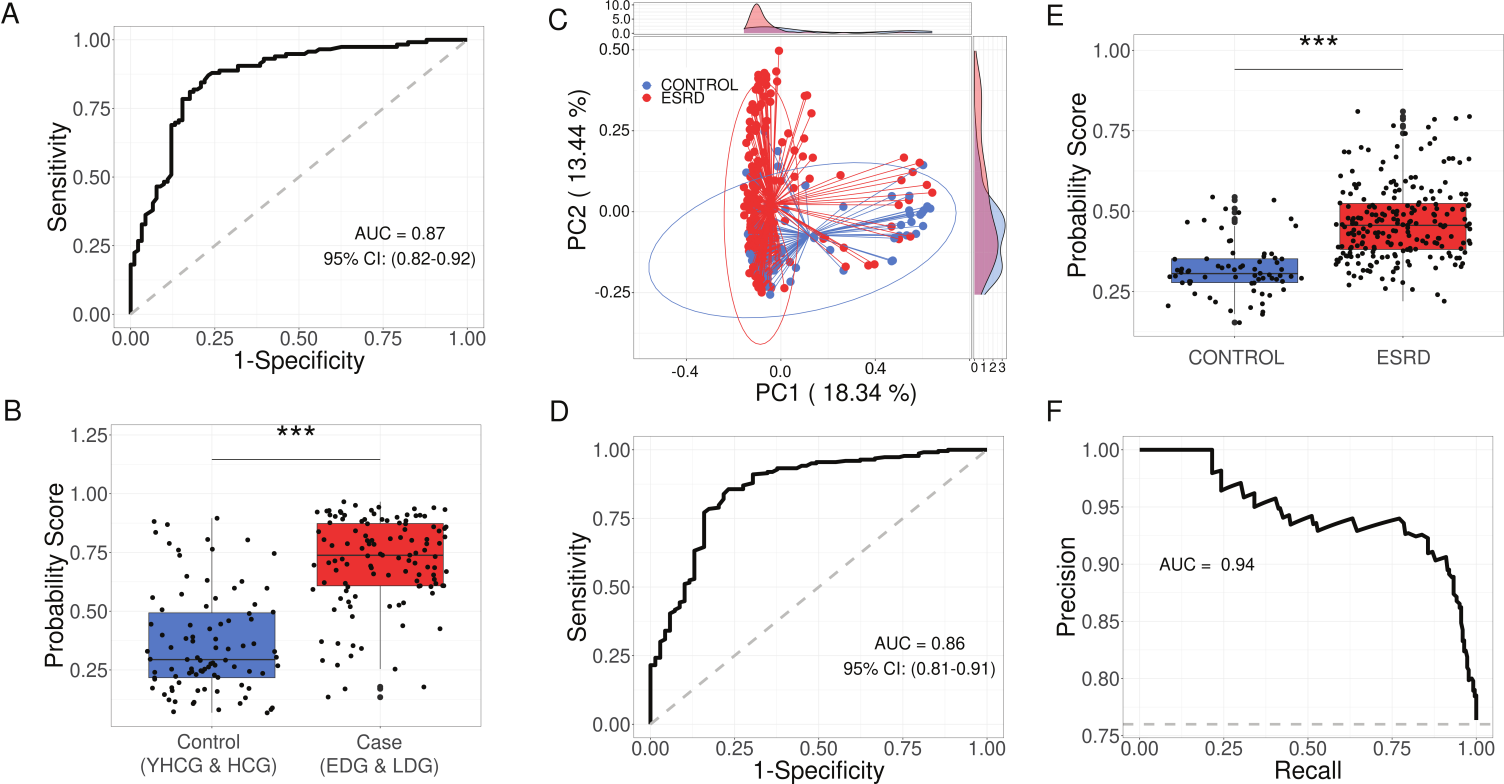
Microbial members of the two guilds as microbiome signature for kidney diseases. (**A**) Based on the abundance matrix of the 54 HQMAGs, a random forest classification model with leave-one-out cross validation was trained to classify control (YHCG and HCG) vs case (EDG and LDG). The AUROC is shown here. (**B**) Box plot of the probability score between control (YHCG and HCG) and case (EDG and LDG). Mann-Whitney test was applied. ***P < 0.001. Control *n* = 91 and case *n* = 116. (**C**) PCoA based on Bray-Curtis distance calculated from the 54 HQMAGs in the validation data set. (**D**) Classify control and ESRD by using the random forest classifier trained in panel **A**. Classifier performance assessed by AUROC. (**E**) Box plot of the probability score between control and ESRD samples in the validation data set. Mann-Whitney test was applied. ***P < 0.001. Control *n* = 69 and ESRD *n* = 223. (**F**) Classify control and ESRD by using the random forest classifier trained in panel **A**. Classifier performance assessed by AUPRC.

To determine the potential of the genomes in the two guilds as general biomarkers for kidney disease, we initially utilized the 54 HQMAGs from guild 1 and guild 2 to construct a random forest classifier. This classifier aimed to distinguish between controls (YHCG and HCG) and cases (EDG and LDG) within our data set (Fig. 6A), achieving notable performance with an AUROC value of 0.87. The model produced a probability score reflecting the likelihood of sample classification as a case, which was significantly elevated in cases compared to controls (Fig. 6B). Subsequently, we accessed metagenomes from an independent study that comprised 69 healthy controls and 223 hemodialysis patients with ESRD (29). Wang et al. categorized the 223 ESRD patients into three groups: glomerulonephritis (*n* = 76), diabetic nephropathy (*n* = 83), and other (*n* = 74) (29); however, this stratification was not utilized here due to the absence of such information in the original publication and online materials. In this external data set, we employed the 54 HQMAGs for read-recruitment analysis to estimate the abundance of reference genomes within the metagenomes. On average, these 54 HQMAGs represented 13.59% ± 8.45% (mean ± SD) of the total gut microbial community abundance. Beta-diversity analysis, based on Bray-Curtis distances, revealed a significant difference in the microbiome signature between the control and ESRD groups (Fig. 6C; PERMANOVA test *P* = 0.001). Introducing the abundance of the 54 HQMAGs into the Random Forest classifier, trained on our data set, to differentiate control and ESRD patients resulted in an AUROC of 0.86 (Fig. 6D). The probability scores were notably higher in the ESRD group compared to the control group (Fig. 6E). Considering the sample size disparity between the ESRD and control groups, we calculated the area under the Precision-Recall curve (AUPRC), achieving an AUPRC of 0.94 with a baseline of 0.76 (Fig. 6F). These findings demonstrate the efficacy of our Random Forest classifier as a diagnostic tool for kidney diseases, indicating that the microbiome signature identified in our study could be a prevalent feature in kidney disease progression.

## DISCUSSION

In this study, we identified two bacterial guilds composed of 54 HQMAGs that are closely associated with the severity and complication risk of DKD patients. Based on these 54 HQMAGs, we further validated this guild-based microbiome signature in an independent research data set of ESRD.

First, to identify key pathogens and beneficial bacteria potentially involved in the development of DKD, our study adopted a genome-centric, guild-based microbiome analysis strategy. Most previous observational results rely on existing databases to classify members of microbial communities into different taxonomic units and explore their correlations with DKD (22, 23). Our method, utilizing *de novo*-assembled HQMAGs from metagenomic data sets as sequence tags, enables us to analyze microbiome data with a resolution far superior to species or any higher taxonomic unit. This genome-centric analysis strategy overcomes two limitations of data analysis based on taxonomic units (24). The first limitation is the reliance on databases for taxonomic annotation, which excludes unclassifiable unknown microbial sequences from further analysis. The second is the aggregation of sequences within the same taxonomic unit that may have different or even opposite relationships with the disease, causing information distortion. Our genome-centric analysis is not limited by existing databases; we combine functionally similar microbes into guilds according to co-abundance behavior and then determine their relationship with health based on the correlation of guilds with host clinical indicators.

Previously published studies based on taxonomic units found that in DKD, the abundance of *Escherichia* increased, along with a decrease in *Bifidobacterium* (21). In our study, a total of 14 *Escherichia* and 69 *Bifidobacterium* HQMAGs were assembled in our data set, but only two strains of *E. coli* and one *Bifidobacterium dentium* were significantly enriched in LDG, which were positively correlated with disease severity, indicating that not all the strains were associated with the severity of DKD, and there may be inconsistent relationships between different strains within the same taxon. Previous studies have found that *F. Prausnitzii*, a short-chain fatty acid-producing bacterium, is reduced in abundance in patients with chronic kidney disease and involved in disease progression (28). However, in our results, only one out of five *F. Prausnitzii* HQMAGs was reduced, indicating that the association between the gut microbiota and DKD is strain- and genome-specific. Therefore, studies at the species or other taxon level alone cannot provide a more favorable solution for researching the relationship between gut microbiota and DKD.

Second, we analyzed the microbiome features involved in the development of DKD not only at the genome level but also further established a genomic ecological network significantly correlated with the primary observational indicator eGFR. Previous researchers established random forest models in DKD and non-DKD by 16S rRNA sequencing but chose 10 key operational taxonomic units (OTUs) related to clinical indicators from non-differential bacteria such as *Lactobacillus* and *Lachnospiraceae* as biomarkers for the diagnosis of DKD (22). Our results show that these genomes are enriched in the two guilds, a potentially beneficial guild 1 and a potentially harmful guild 2. This type of network structure with two guilds is considered a core microbiome feature associated with various chronic diseases (29). Compared with guild 2, guild 1 has a higher genetic capacity for SCFA production, while guild 2 contains more genes encoding uremic toxins (indole biosynthesis), virulence factors, and antibiotic resistance genes. Previous literature has reported that the SCFA levels in DKD patients significantly decrease, and fecal and serum acetate levels positively correlate with eGFR levels (30). The main effects of SCFAs on kidney functions include decreasing inflammation and enhancing antioxidant activity (31). Reduction of inflammation can reduce serum creatine and blood urea nitrogen to improve renal function. Enhancement of antioxidant activity can lead to the decline of renal fibrosis and amelioration of tubular damage. Bacteria carrying VF- and ARG-encoding genes are significantly increased in patients with chronic kidney disease and kidney transplant (32, 33). It has also been reported that patients with impaired renal function have a higher risk of infection by multidrug-resistance bacteria (34). Compared with guild 1, guild 2 uniquely encoded several efflux pumps related to multidrug resistance such as Abc and Mex. VFs are defined as gene products that enable bacteria to colonize, proliferate, and cause damage to the host via both direct and indirect ways (35). A remarkably higher copy of VFs, such as those involved in LPS production and effector delivery system, in guild 2 suggests its higher risk to the host. In-depth investigations into the role of the differential genes between the two guilds in the development of DKD are worth conducting in the future. Our findings that a higher abundance of guild 2 and a lower abundance of guild 1 can distinguish between DKD and healthy controls and reflect the severity of the DKD. Our study identified key bacterial groups containing genomes with genetic capacity to produce harmful metabolites such as uremic toxins through functional gene annotation analysis, providing valuable evidence for discovering new DKD biomarkers and subsequent identification and isolation of key bacterial strains for pathogenesis research.

In addition, the microbial-based classifier trained on our data set effectively discriminates between ESRD patients and healthy controls in an external validation cohort. ESRD has a complex etiology, with DKD being the most common cause (36); therefore, the model shows good discrimination for end-stage renal diseases of various etiologies, suggesting that utilizing guild-based microbiome features has strong universality in the identification of kidney diseases.

The potential mechanisms of how the two guilds may affect DKD initiation and progression warrant further studies. The gut barrier interacts with the gut immune system to maintain intestinal homeostasis, with the gut microbiota playing a significant role in preserving the integrity of the intestinal epithelium and barrier (37). Numerous studies indicate that a dysregulated microbiota can impair intestinal barrier function, allowing potentially pathogenic bacteria and their antigens, such as endotoxins, to translocate into the systemic circulation (16, 38). This can induce systemic and localized immune inflammation through pathways such as reduced production of metabolites like SCFAs (39) and increased accumulation of gut-derived toxins (40), exacerbating kidney damage. In our study, we found that the microbiome features of the two guilds are related to the clinical indicators used to assess the severity of DKD, including eGFR, UACR, CREA, CysC, RBP, HbA1c, and LDH (41, 42), which are also important clinical indicators for predicting the progression of DKD and the risk of cardiovascular disease (CVD) or all-cause mortality (41, 43–46). Serum ALB is the main carrier protein for protein-bound gut-derived uremic toxins (PBUTs), making the toxin molecules difficult to clear from the body and cause kidney damage (47). PBUTs can further alter serum albumin structure, blocking binding sites for other valuable endogenous or exogenous substances (48), and thus may result in a decline of free serum albumin with the aggravation of renal injury. Additionally, with the progression of the disease to the end stage, persistent hypoproteinemia can be caused by recurrent infection, anemia, malnutrition, and so on. The above results all indicate that guild-based microbiome features could become early predictive factors for the risk of DKD, as they are significantly positively correlated with clinical indicators of adverse outcomes, especially those carrying specific functional genes, providing an important foundation for establishing new biomarkers and subsequent targeted interventions for DKD in clinical practice.

Our study is limited by the size and geographical distribution of the study population. The gut microbiota is influenced by regional environments and lifestyles; therefore, multi-center, large-sample population cohort studies are needed for further validation. This project is based on a genome-level exploration of key functional bacterial groups involved in the development of DKD, and further isolation and mechanism studies are pending. Our team has previously conducted a clinical randomized controlled study in patients with diabetic peripheral neuropathy (49), where transplanting healthy donor microbiota to patients increased the abundance of the SCFA-producing guild 1, reduced the more endotoxin-synthesis gene-containing guild 2, improved intestinal barrier integrity, lowered pro-inflammatory cytokine levels, and effectively relieved clinical symptoms. This suggests that such mutually competitive guild-based microbiome features may have targeted therapeutic potential (49), also providing possibilities for future interventions targeting the gut microbiota in DKD patients, especially those with severe disease.

With a genome-centric and guild-based microbiome analysis strategy, we discovered two functional groups in DKD patients, the imbalance of which is closely related to the occurrence and development of DKD. The analysis strategy of this study is unaffected by databases, avoids spurious variables, and can accurately and effectively capture functional groups of gut bacteria relevant to DKD. Our study opens a new avenue for early disease identification and future targeted microbiome therapy for DKD.

## MATERIALS AND METHODS

### Subject recruitment and sample collection

This study was conducted at Henan Provincial People’s Hospital. A total of 207 participants, including 91 healthy controls who visited the hospital for their annual physical examination and 116 DKD patients, were recruited. All DKD patients were divided into two groups based on eGFR: the early-stage DKD (EDG; eGFR ≥ 60 mL/min/1.73 m^2^) and the late-stage DKD (LDG; eGFR < 60 mL/min/1.73 m^2^). All healthy controls conclude two subgroups: HCG) and YHCG. The clinical data of the study subjects, including patient epidemiology (age and gender) and respective clinical laboratory test results (clinical chemistry and metabolic indices), were stored in a computerized database in the hospital medical record system. All participants underwent an overnight fast of no less than eight frozen immediately at −80°C until processing.

The DKD patients were recruited if they (i) were diagnosed by senior clinicians in strict accordance with the criteria of the clinical guideline for prevention and treatment of diabetic kidney disease in China (2021 edition) issued by the Chinese Medical Association; (ii) age of 18–70 years; (iii) 6.5% ≤ HbA1c ≤ 11%. The following exclusion criteria were applied to all groups: (i) had a continuous antibiotic use history for >3 days within 3 months prior to enrollment; (ii) had renal injury considered likely to be the non-diabetic kidney disease: a history of diabetes less than 5 years with massive proteinuria or renal insufficiency, a short period of massive proteinuria or nephrotic syndrome, and unexplained rapid decline in eGFR; had malignancy or infectious diseases; (iii) the blood pressure was still ≥180/110 mmHg after drug treatment; (iv) had undergone gastrectomy, fundoplication, colostomy, or other digestive system surgery; (v) had persistent vomiting or a suspected gastrointestinal obstruction; (vi) had severe cardiovascular and cerebrovascular diseases or liver, kidney, and hematopoietic system diseases; (vii) had alcoholism (drinking more than five times in 1 week, more than 100 g of spirits, 250 g of rice wine, or five bottles of beer); or (viii) were pregnant.

### Clinical laboratory examination and data collection

All laboratory tests were conducted at the clinical laboratory of Henan Provincial People’s Hospital. Biochemical parameters, such as CREA, UA, CysC, LDL-C, HDL-C, TG, CK, ALT, AST, ALB, and LDH, were measured by a biochemical immunoassay workstation (Aeroset, Abbott Laboratories Co., USA). eGFR was further calculated using CKD-EPI (http://www.nkdep.nih.gov). UACR was measured by the automatic specific proteins analyzer (BA400, Biosystems Co., Spain). Plasma HbA1c concentrations were measured by automatic high-performance liquid glycated hemoglobin analyzer (H9, Lifotronic Co., China).

### Gut microbiome analysis

#### DNA extraction and metagenomic sequencing

Genomic DNA was extracted from human feces using the Hipure Stool DNA Mini kit (D3141). Next-generation sequencing library preparations were constructed following the manufacturer’s protocol. Two hundred microgram of genomic DNA was randomly fragmented by Covaris to an average size of 300–350 bp. The fragments were treated with End Prep Enzyme Mix for end repairing and 5′ phosphorylation and 3′ adenylated, to add adaptors to both ends. Size selection of adaptor-ligated DNA was then performed by DNA cleanup beads. Each sample was then amplified by PCR for eight cycles using P5 and P7 primers, with both primers carrying sequences that can anneal with flowcell to perform bridge PCR and P7 primer carrying a six-base index allowing for multiplexing. The PCR products were cleaned up and validated using an Agilent 2100 Bioanalyzer. The qualified libraries were sequenced pair end PE150 on the illumina Novaseq6000 System.

#### Data quality control

KneadData (https://huttenhower.sph.harvard.edu/kneaddata/) was applied to perform quality control of the raw reads with the following parameters decontaminate-pairs strict, -run-trim-repetitive, -bypass-trf, and -trimmomatic-options = “slidingwindow:4:20 minlen:60.” Reads that could be aligned to the human genome were identified and removed in KneadData by aligning reads against the *Homo sapiens* hg37 genome.

#### High-quality metagenome-assembled genome construction, abundance calculation, and taxonomic assignment

High-quality reads were assembled into contigs in each sample by using MEGAHIT (-min-contig-len 500 and -preset meta-large) (50). The assembled contigs were binned with MetaBAT 2 (51) and MaxBin 2 (52). A refinement step was then performed using the bin_refinement module from MetaWRAP (53) to combine and improve the results generated by the two binners. The quality of the bins was assessed using CheckM2 (54). Bins with completeness >95% and contamination <5% were retained as high-quality draft genomes. The assembled high-quality draft genomes were further dereplicated by using dRep (55). DiTASiC (56), which applies kallisto for pseudo-alignment (57) and a generalized linear model for resolving shared reads among genomes, was used to calculate the abundance of the genomes in each sample, and estimated counts with *P* > 0.05 were removed. GTDB-Tk (database v214.1) (58) was used to conduct taxonomic assignment of the genomes.

#### Functional analysis of the genomes

Genomes were annotated using Prokka (59). We used KofamKOALA (KEGG release 109.0) to assign KO to the predicted protein sequences in each genome (60). Genes encoding formate-tetrahydrofolate ligase, propionyl-CoA:succinate-CoA transferase, propionate CoA-transferase, 4Hbt, AtoA, AtoD, Buk, and But were identified as described previously (19). Antibiotic resistance genes were predicted using ResFinder (61) with the default parameters. Identification of virulence factors was based on the core set of the Virulence Factors of Pathogenic Bacteria Database (VFDB [62]; downloaded on January 2024). Predicted protein sequences were aligned to the reference sequence in VFDB using BLASTP (best hist with E value < 1e−5, identity > 80%, and query coverage > 70%)

#### Co-abundance network construction and analysis

Fastspar (63), a rapid and scalable correlation estimation tool for microbiome study, was used to calculate the co-abundance correlations between the genomes with 1,000 permutations across all samples. Complete linkage based on the co-abundance correlations followed by WGCNA (25) was used to identify the two guilds.

#### Validation in an independent cohort

Metagenomic sequencing data from 69 healthy controls and 223 hemodialysis patients who were diagnosed with ESRD were from the European Nucleotide Archive database under PRJNA449784. Data quality control was performed as described in “Data quality control” above. The abundance of the 54 genomes was calculated by DiTASiC as described before.

### Statistical analysis

Statistical analysis was performed in R version 4.2.1. A Kruskal-Wallis test followed by Dunn’s post hoc test (two-sided) was used to compare the different groups. Redundancy analysis was conducted based on the Hellinger transformed abundance to find specific gut microbial members associated with group assignment. Both single-factor and marginal PERMANOVA tests including both age and group assignment were used to compare overall gut microbial composition. AUROC was used to evaluate the capacity of microbiome signatures to discriminate between groups using the R package pROC. AUROC considers the trade-offs between sensitivity and specificity and compares the performance of classifiers with a baseline value of 0.5 for a random classifier. Due to the imbalance in the sample size between control and ESRD in the validation data set, AUPRC, applied via PRROC (64), was used to assess the performance of classification as well. AUPRC, which considers the trade-offs between precision and recall with a baseline that equals the proportion of positive cases in all samples, was used as a complementary assessment, particularly for highly imbalanced data sets.

## Data Availability

The metagenomic sequencing data for the current study have been deposited into the CNGB Sequence Archive (CNGB) of the China National GenBank Database (CNGBdb) (65) under accession no. CNP0005367. Parameters of the bioinformatic tools applied in the study are listed in Materials and Methods. Scripts and command lines related to the current study can be found at https://github.com/nightkid03/DKD.

## SUPPLEMENTAL MATERIAL

Supplemental material mbio.00735-24-s0001.docx Fig. S1–S3.

ASM does not own the copyrights to Supplemental Material that may be linked to, or accessed through, an article. The authors have granted ASM a non-exclusive, world-wide license to publish the Supplemental Material files. Please contact the corresponding author directly for reuse.
