# Supplementary material for: Guild-level signature of gut microbiome for diabetic kidney disease
Source: mBio. 2024 May 31;15(7):e00735-24. doi: 10.1128/mbio.00735-24 (PMC11253615; doi:10.1128/mbio.00735-24)
Supplement: Supplemental material — Fig. S1–S3. [file mbio.00735-24-s0001.docx]

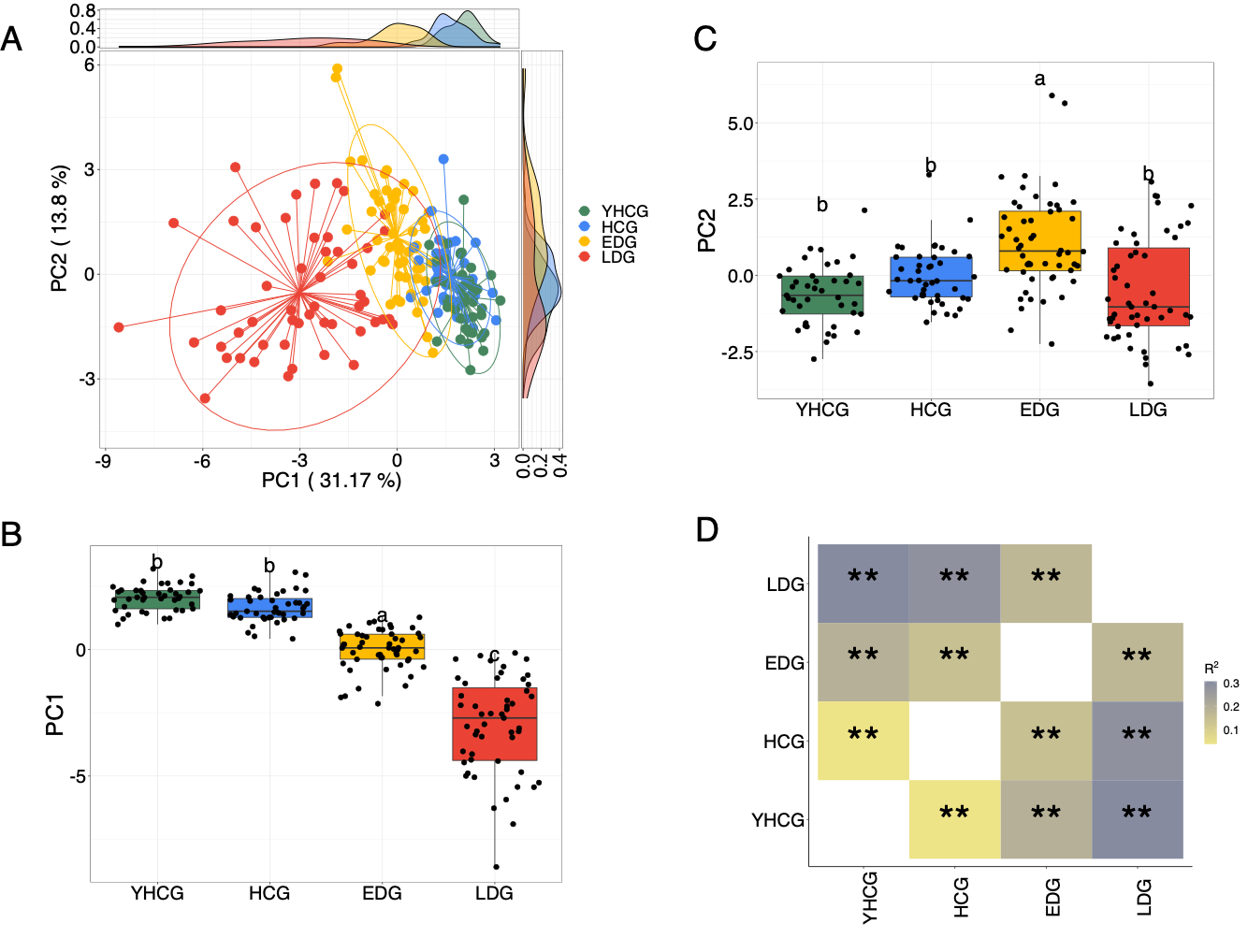


**Figure S1. Difference in the clinical parameters between the groups.** (A) Principal Coordinate Analysis (PCoA) based on the Euclidean distances calculated from z-score transformed clinical parameters in table 1, illustrating the distribution and clustering of the participants among the study groups. (B) & (C) Comparative analysis of the PC1 and PC2 scores across the groups. Box plots represent the median values and interquartile ranges (IQRs), with whiskers extending to the lowest and highest values within 1.5 times the IQR from the first and third quartiles, respectively. Outliers are depicted as individual points. The Kruskal-Wallis test followed by Dunn’s post hoc test (two-sided) was employed for group comparisons. Compact letter displays denote the significance as per the post hoc test, with Benjamini-Hochberg (BH) adjusted P-values: P < 0.05 deemed significant. (D) Pairwise PERMANOVA testing among the four groups, with ** indicating BH adjusted P < 0.01. The groups are young healthy control group (YHCG, n = 45), healthy control group (HCG, n = 46), early-stage DKD (EDG, n = 57), and late-stage DKD (LDG, n = 59).

**Figure S2. Tri-plot of redundancy analysis (RDA) of the microbial composition based on the 1,543 high quality metagenome-assembled genomes (HQMAGs).** The group assignment was used as environmental variables. Samples are indicated by dots. HQMAGs with at least 10% of the variability in their abundance explained by RDA1 and RDA2 are indicated by blue arrows. RDA analysis was conducted based on the Hellinger transformed abundance

**Figure S3. Venn diagram shows the functional differences between the two guilds at module level.** In each guild, the KOs predicted from the genomes were mapped to KEGG modules.
